# Intra-individual Genomic Variation Analysis in Tissues (Blood *vs.* Testis) Through SNP Microarray: A Case Report of Two Patients with Idiopathic Sertoli Cell Only Syndrome (SCOS)

**DOI:** 10.18502/jri.v21i4.4335

**Published:** 2020

**Authors:** Aiyush Sharma, Ashutosh Halder, Seema Kaushal, Manish Jain

**Affiliations:** 1- Department of Reproductive Biology, All India Institute of Medical Sciences, New Delhi, India; 2- Department of Pathology, All India Institute of Medical Sciences, New Delhi, India

**Keywords:** Copy number variations, Loss of heterozygosity, Sertoli cell only syndrome, Single nucleotide polymorphisms

## Abstract

**Background:**

Sertoli cell only syndrome (SCOS) or germ cell aplasia is characterized by the existence of only sertoli cells in the seminiferous tubule without any germ cells. SCOS is a multifactorial disorder but genetic factors play a major role in pathogenesis of idiopathic SCOS.

**Case Presentation:**

Two cases of idiopathic SCOS had been reported with no non-genetic factor in their medical history that could play a role in aetiology of SCOS. Also, two normal fertile males were recruited as controls in this study. For evaluation of genomic imbalance, karyotyping (G-banding), FISH, STS-PCR and SNP microarray were carried out. SNP microarray was carried out in DNA of peripheral blood for cases as well as controls. However, for cases, SNP microarray was conducted in DNA of testicular Fine needle aspiration cytology (FNAC).

**Conclusion:**

No chromosome abnormality and Yq microdeletion was found in cases as well as in controls. Microarray detected many CNVs and LOH that cover genes with spermatogenesis related function and PAR CNVs in both cases. Differential genomic variations were found in blood and testis for cases. Therefore, the evaluation of pathogenesis of idiopathic SCOS might be dependent on both tissue samples. The evaluation of genomic imbalances at both tissue levels should be done for a large cohort of patients.

## Introduction

Sertoli cell only syndrome (SCOS) is characterized by the existence of only sertoli cells in the seminiferous tubule and without any germ cells. SCOS is one of the reasons for azoospermia in infertile men. It is also called Del Castillo syndrome or germ cell aplasia. According to Del Castillo et al. (1947), SCOS has the following characteristics: 1) Normal secondary sexual characteristics, 2) Absence of germ cells in seminiferous tubule and, 3) Less severe histological degeneration in testis (1). It represents a subset of NOA that shows absence of all spermatogenic lineages of cells in testicular biopsy or FNAC samples.

Spermatogenesis is a complex process that produces terminally differentiated spermatozoa from spermatogonial stem cells (SSCs) within seminiferous tubule. This process starts in fetal life when male germ cells undergo mitotic arrest before entering into the proliferative and differentiating phase at puberty. SSCs have two fates; either they maintain stem cell identity or they become terminally differentiated into sperm. There must be a balance between SSCs self renewal and differentiation for sustaining spermatogenesis and male fertility. The balance between these processes requires an intercellular communication between SSCs and their supporting somatic cells, especially sertoli cells. Sertoli cells are a vital part of testicular microenvironment or niche (2). Sertoli cells precisely control the division and differentiation of SSCs. Any imbalance between SSCs self renewal and differentiation completely interrupts spermatogenesis resulting into a testicular phenotype called SCOS (3). Therefore, the focus of this study was to investigate any genomic abnormality in the sertoli cells which results in germ cell loss.

SCOS patients have no germ cells, so follicle-stimulating hormone (FSH) levels are usually high while testosterone and luteinizing hormone (LH) levels are normal in such patients (4).

SCOS is a multifactorial disorder and factors responsible for the anomalous condition are cryptorchidism, varicocele, hydrocele, mumps orchitis, testicular trauma, chemotherapy, radiotherapy, cytotoxic drugs, *etc* (5). Underlying causes of approximately 70% cases of this disorder remain largely unclear but some iatrogenic or non-iatrogenic causes are known. The most established cause of SCOS is genetic factor which includes chromosomal abnormality and Y-chromosome microdeletion (Azoospermia factor, AZF) (6). Chromosomal abnormalities account for only 15–20% of azoospermia including SCOS cases (7). Tiepolo et al. (1976) first hypothesized the role of Y chromosome deletions in male infertility (8). Yq microdeletions affect around 10–15% of azoospermic men and 5% of oligospermic men with testicular histologies varying from hypospermatogenesis to maturation arrest or sertoli cell-only syndrome (9).

Chromosomal rearrangements causing deletions, duplications, or unbalanced translocations result in inappropriate gene dosage. High-resolution microarray of autosomes and sex chromosomes has detected many Copy Number Variations (CNVs) in infertile men. This indicates the need for studying sex chromosomes to investigate azoospermia (Especially non-obstructive azoospermia). CNVs are responsible for inappropriate gene dosage, alteration in gene function by interrupting gene, creating fusion gene, activation of recessive mutation and altering position effect of genes (10, 11). Chromosomal rearrangements also include allelic imbalance in segments identified as loss of heterozygosity (LOH) at polymorphic loci. LOH implicates regions harbouring genes with functions related to spermatogenesis. The SNP array provides a single platform for genome wide analysis of copy number variations, LOH, microdeletions, microduplications and mosaicism. In this study, an attempt was made to explore the genes with the known functions related to spermatogenesis and also some novel genes that may be related to spermatogenesis using microarray.

Genetic variation between individuals has been extensively investigated, but intra-individual genomic variation among different tissues is not well studied. It is presumed that all the cells originated from the same zygote possess the same genomic content. However, some studies are available that show the intra-individual genomic variation between differentiated tissues. This study investigated the scope of genomic variation between tissues (Blood *vs*. testis) intra-individually.

## Case Presentation

In this study, 4 *ml* of clotted blood was collected for hormone evaluation (FSH, testosterone and AMH). Next, 3 *ml* of EDTA blood for DNA and interphase cell preparation besides 1 *ml* of heparinized blood for metaphase cell preparation was utilized. Testicular fine needle aspiration cytology (FNAC) samples were used for evaluation of patients DNA.

### Recruitment:

Two cases who referred to the Department of Reproductive Biology, AIIMS, New Delhi in July, 2018 were the subjects of the study. They were concerned about infertility due to azoospermia. Semen analysis in both cases revealed azoospermia on three occasions after more than 3 months of interval and following 2–5 days of abstinence. Their medical history, secondary sexual characteristics and testis size were documented. Medical history was negative for diseases which could have been responsible for germ cell loss in both the cases. Their secondary sexual characteristics and testicular size were also normal. FNAC samples were collected from both the patients. Two controls with normal semen parameters were also recruited in this study.

### Hormonal Evaluation:

The hormonal analysis of FSH and testosterone was done in CRIA facility at AIIMS, New Delhi using Abbott ARCHITECT assay kit. AMH was evaluated by ultra-sensitive ELISA kit (Hans’s lab).

### Karyotyping:

Karyotyping was done by Giemsa (G) banding technique on metaphase cells for both cases and controls. Karyotyping was performed to identify numerical and structural chromosomal aberrations (Structural aberration includes deletion, duplication or translocation). Karyotyping slides were analyzed manually as well as by cytovision software.

### Fluorescence in-situ hybridization (FISH):

Fluorescence in situ hybridization (FISH) was done to detect the presence of numerical sex-chromosomal aberrations. Both cases and controls were subjected to FISH for evaluation of sex chromosome aneuploidy/mosaicism by using probes for centromeric region of X and Y chromosome (Our lab made the research probe).

### Multiplex STS-PCR:

Multiplex STS-PCR was done in peripheral blood DNA for screening Yq microdeletion (Structural aberration). DNA was extracted from peripheral blood by Miller’s method. Basic set of 6 STS-primers was used, for monitoring deletion in long arm of Y chromosome. These STS-primers cover 3 AZF-loci, *i.e*., a, b and c. Basic set of 6 STS-primers includes:

AZFa- sY84, sY86
AZFb- sY127, sY134
AZFc- sY254, sY255

These 6 STS-primers were suggested by Simoni et al. in 1999 (12) which detect ~90% deletion in AZF loci. Also, a primer pair of SRY gene was used as an internal control. PCR products were analyzed on 4% agarose gel containing ethidium bromide (10 *mg/ml*).

### SNPs Microarray:

SNP microarray analysis (750 *k*, affymetrix platform) was done for both patients and controls. DNA was extracted from peripheral blood in both cases and controls by Miller’s method. DNA was also extracted from testicular FNAC samples (Bilateral testis) by Qiagen kit from cases only. Data was analyzed using Affymetrix ChAS software.

## Results

Testicular FNAC reports of both patients were positive for SCOS. At first, serum value of FSH, testosterone and AMH was evaluated. Normal serum levels of these 2 hormones were detected in both cases and controls. However, FSH level was borderline in cases only. Details of hormones level are summarized in table 1.

**Table 1.** Details of hormone levels in cases and controls

	**FSH (*mIU/ml*)**	**Testosterone (*ng/ml*)**	**AMH (*ng/ml*)**
**Controls DBN**			
1	2.08	6.88	22.1
2	5.81	2.19	9.1
**Patients DBN**			
27	10.84	6.42	8.19
28	10.28	5.39	3.08

**Figure 1.**
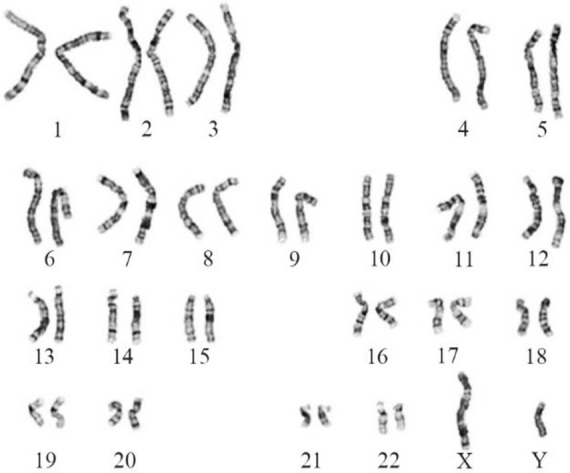
Karyotype image showing normal 46,XY chromosome spread in SCOS case

Karyotyping was done on metaphase cells to identify the numerical and structural chromosomal aberrations. No numerical and structural chromosomal abnormalities were found in cases as well as controls. Karyotype image is shown in figure 1.

**Figure 2.**
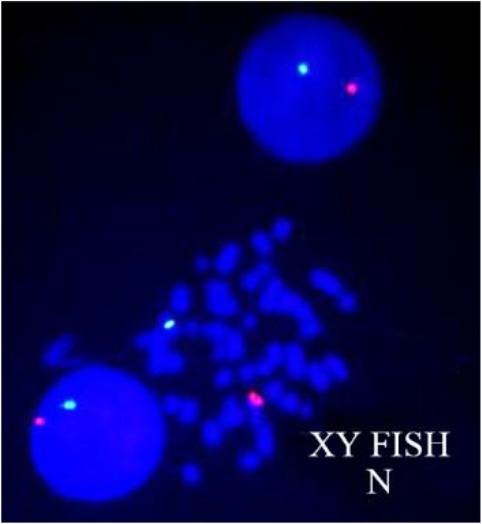
XY FISH image showing one green (X-centromere) and one red (Y-centromere) signal in interphase and metaphase cells of patient indicating normal (N) male sex chromosome

**Figure 3.**
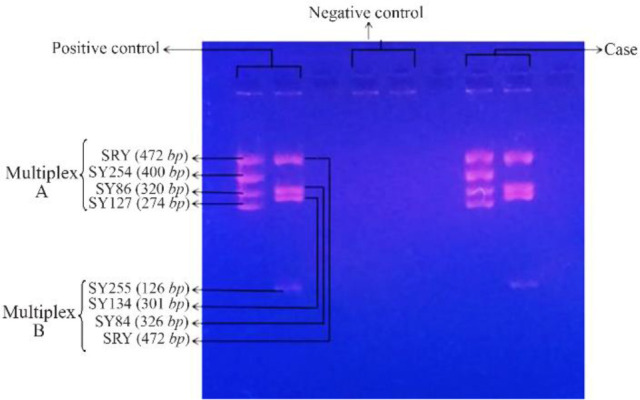
Multiplex-STS PCR picture showing no deletion in AZF region in both positive control (left panel) and SCOS case (right panel). No AZF region was present in negative control (middle panel)

Patients and controls were then subjected to various molecular investigations such as FISH, STS-PCR and SNPs microarray. FISH was done with centromeric probes of X and Y chromosomes on interphase cells as well as metaphase cells for determining numerical aberrations of sex chromosomes. FISH confirmed the result of karyotyping. Both cases and controls showed no sex chromosome abnormalities. XY FISH image is shown in figure 2. Further, cases and controls were analyzed for Yq microdeletion (Structural aberration) by multiplex STS-PCR using the basic set of 6 primers. No deletion in AZF loci was detected by multiplex STS-PCR. Image of multiplex STS-PCR showing no deletions of AZF loci in agarose gel (4%) is shown in figure 3.

Finally, genomic imbalances were analyzed by SNPs microarray in different tissues (Blood *vs*. testis) to check intra-individual genetic variations in cases only. Also, SNP microarray analysis was done in both controls on blood DNA only. Microarray findings in patients were compared with controls’ microarray data and online CNVs database (http://dgv.tcag.ca/dgv/app/home?). It was found that LOH and CNVs genes are related with 1spermatogenetic function and CNVs cover pseudoautosomal region (PAR). Microarray detected 3 autosomal LOH regions and 1 CNV in the DNA of testicular tissue in one patient (database number 27 or DBN 27). However, in the DNA of peripheral blood of DBN 27 patient, microarray detected 3 autosomal LOH regions and 2 CNVs.

In the case of DBN 28 patient, 4 autosomal LOH regions and 1 CNV in DNA of testicular tissue were detected while in peripheral blood DNA, only 4 autosomal LOH regions were detected. All data is summarized in table 2. Genes covered by CNVs/LOH were annotated for spermatogenesis related functions from OMIM and UniProt. Genes’ function related to spermatogenesis is presented in table 3.

**Table 2.** Comparison of CNVs/LOH detected in two patients’ peripheral blood DNA and DNA of testicular tissue with controls

**Patients’ DBN**	**Sample**	**Region**	**Start**	**End**	**Size (*kb*)**	**Type**	**Genes**	**Controls having CNV/LOH**
**27**	Tissue (Rt. & Lt. testis)					LOH	CELF1	None
						LOH	CADM4, CATSPERG, DMRTC2, ETV2, GAPDHS, GGN, LIPE, TEX101	None
		Yq11.221	16165256	16285871	120.615	Gain	VCY, VCY1B	None
						LOH	CELF1	None
	Peripheral blood	11q12.3-q13.2	62755497	67833543	5078.046	LOH	OVOL1, CCDC87, CATSPER1, HRASLS5, PLA2G16, TEX40	None
		Xp22.33	2367576	2392209	24.633	Loss	DHRSX	None
						Gain	VCY, VCY1B	None

**28**	Tissue (Rt. & Lt. testis)	3p21.31-p21.1	46570985	52595417	6024.432	LOH	NDUFAF3, PRKAR2A, ZMYND10, CDC25A, DNAH1, IQCF1, PHF7	None
							NKAPL, TRIM27, GPX5	None
		10p13-p12.1	21036399	24688985	3652.586	LOH	SPAG6	None
						LOH	BNC1	None
		Xp22.33	2703822	2740608	36.786	Gain	XG	None
	Peripheral blood					LOH	NDUFAF3, PRKAR2A, ZMYND10, CDC25A, DNAH1, IQCF1, PHF7	None
		6p22.2-p22.1	26374548	29537975	3163.427	LOH	NKAPL, TRIM27, GPX5	None
						LOH	SPAG6	None
		15q25.1-q25.3	81488369	88927748	7439.379	LOH	BNC1	None

**Table 3.** Genes’ function related to spermatogenesis

**Gene**	**Region**	**Function**	

		**UNIPROT**	**OMIM**
**NDUFAF3**	3p21.31		In situ hybridization detected expression of this gene at stage I pachytene spermatocytes, and expression persisted upto stage XII of the spermatogenic cycle in mouse testis (13)
**PRKAR2A**	3p21.31		PRKAR2A expression is localized in sperm flagella and does phosphorylation necessary for sperm motility. High expression was detected in haploid germ cells (14)
**ZMYND10**	3p21.31	ZMYND10 plays a role in organization of axoneme structure by assembling inner and outer dynein arms and hence controlling motility	In adults, ZMYND10 expression was confined to testis. ZMYND10 mutated mice were infertile. Mutant flies had immotile sperms and flagella showed a partial loss of dynein arms (15)
**CDC25A**	3p21.31		Expression of CDC25A in men with complete spermatogenesis in leptotene spermatocytes for elongating spermatids but no expression in men with spermatogenic failure (16)
**IQCF1**	3p21.2	Regulates sperm capacitation and acrosome reaction	
**PHF7**	3p21.1	May involve in spermatogenesis	
**DNAH1**	3p21.1	Protein of cilia essential for sperm flagellum motility as it involves in formation of the inner dynein arms and axoneme (PubMed: 24360805)	Ben Khelifa et al. (2014) found localization of DNAH1 over the full length of the sperm flagellum in immunohistochemistry
**NKAPL**	6p22.1	Transcriptional repressor that is required for spermatogenesis	
**TRIM27**	6p22.1	E3 ubiquitin-protein ligase that has a transcriptional repressor activity and induces apoptosis. It may function in male germ cell development	
**GPX5**	6p22.1	It may constitute a glutathione peroxidase-like protective system which prevents peroxide damage in sperm membrane lipids	The mammalian epididymis had expression of GPX5 that protects sperm cells from oxidative damage (17)
**SPAG6**	10p12.2	Essential for structural integrity of central apparatus in sperm tail and for flagella motility	Sapiro et al. (2002) found that SPAG6 null mice had infertility because they showed significant motility defects, abnormal sperm morphology and loss of sperm head. So, they concluded that SPAG6 is important for structural integrity of mature sperm (18)
**HRASLS5**	11q12.3		HRASLS5 may regulate cell growth and differentiation in the testis (19)
**CELF1**	11p11.2	RNA-binding protein that is essential for spermatogenesis completion	
**PLA2G16**	11q12.3-q13.1		In situ hybridization detected expression of PLA2G16 to seminiferous tubules in association with round spermatids (20)
**TEX40**	11q13.1	Component of Catsper complex which involved in sperm cell hyperactivation. Hyperactivation is required for sperm motility for fertilization. It is quadrilaterally arranged along flagella	Postnatally, TEX40 mRNA expressed in germ cells before Catsper1 expression. It is localized in principal piece of sperm tail and quadrilaterally arranged along sperm flagella in human and mouse (21)
**OVOL1**	11q13.1	Transcription factor which is considered to be involved in spermatogenesis	
**CATSPER1**	11q13.1	Voltage-gated calcium channel responsible for sperm hyperactivation, acrosome reaction and chemotaxis towards the oocyte	Catsper 1 is a sperm-specific ion channel responsible for calcium entry in sperm for hyperactivated motility and normal male fertility. Immunofluorescence showed Catsper1 expression in principal piece of the sperm tail (22)
**CCDC87**	11q13.2	It plays an important role in normal sperm head morphology during spermatogenesis. It is also essential for acrosome reaction and hence normal male fertility	
**DMRTC2**	19q13.2	May play a role in sexual development	PCR analysis detected strong expression in testis (23)
**TEX101**	19q13.31	It regulates binding of sperm to zona pellucida and migration of spermatozoa in oviduct	It was identified as a testicular germ cell antigen. Immunohistochemistry detected expression on cell surfaces of prospermatogonia and plasma membranes of spermatocytes and spermatids (24)
**LIPE**	19q13.2		Immunocytochemistry showed LIPE localization to elongated spermatids and spermatozoa but not in interstitial cells (25)
**GGN**	19q13.2	It may have a role in spermatogenesis	Lu and Bishop (2003) found GGN expression in germ cells from the late pachytene spermatocyte to the round spermatid stages and no expression in sertoli or leydig cells (26)
**APDHS**	19q13.12	Involves in energy production by regulating the switch between different pathways during spermiogenesis and in the spermatozoa. Hence, it plays a role in sperm motility and male fertility	Welch et al. (2000) found GAPDS expression to the principal piece of the flagellum (27). Miki et al. (2004) found that GAPDHS faulty expression blocks sperm glycolysis in mice. GAPDHS null male mice were infertile due to defects in sperm motility (28)
**CADM4**	19q13.31		CADM4 interacted with 4.1 G protein and both colocalized in sertoli cells in mouse testis. 4.1 G null mice had disrupted sertoli-germ cell interactions which caused male sterility (29)
**ETV2**	19q13.12		De Haro and Janknecht (2005) found strong Etv2 expression in sertoli cells by RT-PCR (30)
**CATSPERG**	19q13.2	Catsper1 plays a role in sperm cell hyperactivation essential for sperm motility in fertilization	Wang et al.(2009) found localization of Catsper G in spermatocytes and spermatids (31)
**BNC1**	15q25.2	Transcriptional activator which is essential for maintenance of spermatogenesis	
**VCY1**	Yq11.221	May play a role in a process in spermatogenesis	VCX/Y gene family member present on Y chromosome having expression mainly in male germ cells (32). Immunohistochemistry of human testis detected VCY expression in nuclei of germ cells of seminiferous epithelium (33)
		May play a role in a process in spermatogenesis	

To enhance the clinical interpretation, the SNP microarray findings were compared with DECIPHER (Open access patient database). The clinical relevance and the findings are summarized below in table 4. The same CNV was found in two patients reported in DECIPHER.

**Table 4.** CNVs of testicular tissue in patients matched with DECIPHER database

**Patient DBN**	**Region**	**Size (*kb*)**	**Start**	**End**	**Clinically significant reference region (From DECIPHER-GRCh37/hg19)**	**Gene**
				2740608	Gain X: 1256608-6413964 (DECIPHER ID-290855) (Male infertility) Likely pathogenic	XG
**28**	Xp22.31 gain	36.786	2703822	2740608	Gain X: 61091-11724962 (DECIPHER ID-277458) (Azoospermia), Uncertain	XG

## Discussion

This study tried to give an insight into genetics of idiopathic SCOS. There were 2 cases and 2 controls recruited for this study. First, the cases and controls were evaluated for FSH, testosterone and AMH levels. Serum values of these hormones were within normal reference range. However, borderline FSH level was reported in both cases which indicated an abnormality in the testis. Abel et al. in 2008 found that sertoli cell specific FSHR deletion in mice caused substantial loss of germ cells and disrupted germ cell meiosis demonstrating the importance of FSH in normal testicular function (34). FSH level has a high predictive value for SCOS (35). Serum level of testosterone is within normal reference depicting that both patients have normal androgenisation. In both cases, low serum AMH values were found which showed that sertoli cells were matured and had no abnormality. Sertoli cells start secreting AMH in male fetus at 8th week and serum AMH level remains high till pre-puberty stage. Puberty in male is marked by substantial decrease in serum AMH indicating sertoli cells maturation (36).

Karyotyping was performed to screen numerical as well as structural chromosomal aberrations in both cases and controls. No sex chromosome aneuploidies were observed in cases and controls by FISH using centromeric probes of X and Y chromosomes. Sex chromosome aneuploidies have a prevalence of around 15% only among infertile men (37). Klinefelter syndrome is the most common numerical sex chromosome abnormality among aneuploidies that influences 10% of men with azoospermia (38). Non-mosaic Klinefelter patients often have SCOS condition or very few germ cells in their testis (39).

Further, analysis for Yq microdeletion was done by multiplex STS-PCR using basic set of 6 primers. It was quoted in Yq microdeletion studies that AZFa region deletion was majorly attributed to testicular phenotype, SCOS, and AZFb deletion caused spermatogenic arrest and AZFc deletion was responsible for hypospermatogenesis, spermatogenic arrest and SCOS (40, 41). But in this study, no AZF deletion was detected by multiplex STS-PCR in cases as well as controls.

SNP microarray analysis was done to enhance the depth of knowledge for genomic imbalances present in SCOS cases. Microarray was done for peripheral blood as well as DNA of testicular tissue in cases to analyze genomic imbalances at both tissue levels. This study focused on CNVs as well as LOH regions in peripheral blood DNA and DNA of testicular tissue. CNVs are responsible for inappropriate gene dosage, alteration in gene function by interrupting gene, creating fusion gene, activation of recessive mutation, altering position effect of genes (10, 11). Loss of heterozygosity (LOH) is a genetic event most commonly found in cancer patients and has a role in loss of somatic wild type alleles in many types of cancer. Principal mechanism of LOH action is through biallelic inactivation of tumor suppressor gene in cancer (42).

The exact pathway by which LOH impairs spermatogenesis is not known. It might be possible that biallelic inactivation of spermatogenesis related genes caused by LOH regions is similar to biallelic inactivation of tumor suppressor gene caused by LOH regions in cancer. In order to assess potential impact of LOH in pathogenesis, only those LOH regions which contained genes with spermatogenesis related function were the focus of this research.

An attempt was made to investigate the difference in genomic imbalances between the tissues (Blood *vs*. testis) of SCOS patients which are responsible for complete spermatogenic failure. Differences in autosomal LOH and CNVs (Both sex chromosome and autosomes) in DNA of both tissues (Blood *vs*. testis) were identified. Genomic variations among tissues in an individual are understudied. But some studies showed the somatic genomic variations among different tissues intra-individually (43). Huallachain et al. in 2012 found somatic variations in 178 tissue- specific CNVs among 33 tissues intra-individually. Also, age related somatic genome variations were identified by SNP microarray (43). It was hypothesized that genomic variation may occur in early development of cells that are active for cell divisions (44). It can be inferred that complete germ cell loss in SCOS cases might have occurred due to early age genomic variation in gonadal tissue during differentiation or when germ cell enters meiosis. Therefore, it is required to do further analysis for identifying genomic variations among different tissues in the same individual.

The studied autosomal LOH and sex chromosome CNVs in this research consisted of genes related to spermatogenesis function. Sex chromosome CNVs covering PAR region were also reported. CNVs of the PAR genes like PLCXD1, ASMT, ASMTL, CD99, DHRSX, XG cause disrupted synapsis due to structural aberrations and result in no XY pairing and male infertility. PAR regions have genes that resist X inactivation for maintenance of dosage compensation (45).

In this study, 3 autosomal LOH and 1 sex chromosome CNV (Yq11.221 gain) were identified in the DNA of testicular tissue of DBN 27 patient, while in the blood DNA of the same patient, microarray detected 3 autosomal LOH and 2 sex chromosome CNVs (Yq11.221 gain and Xp22.33 loss). Autosomal LOH regions are the same in both tissues (Blood and testicular tissue) but different genomic coordinates in LOH region identify additional genes involved in spermatogenesis function. An additional sex chromosome CNV was detected in patient’s blood that involves PAR1 (Xp22.33) region. PAR1 region deletion in blood DNA and difference of autosomal LOH coverage region in DNA of both tissues might play a role in pathogenesis of SCOS.

In DBN 28 patient, 4 autosomal LOH and 1 sex chromosome CNV (Xp22.33 gain) were found in DNA of testicular tissue by microarray analysis while in blood only 4 similar autosomal LOH regions were detected. Autosomal LOH regions were the same in both samples (Blood and testicular tissue) with almost similar genomic coordinates covered by LOH. An additional sex chromosomal CNV was found in DNA of patient’s testicular tissue that involved PAR1 (Xp22.33) region. Moreover, 7 sex chromosome CNVs [*e.g*., Xp21.2, Xq13.1, Xq13.3, Xq23, Xq26.3, Xq28, Yp11.2] were identified in DNA of testicular tissue but not in DNA of blood. This additional sex chromosome CNV might cause germline loss and hence consequently SCOS in the patient.

Also, these patients’ microarray data was analyzed from DECIPHER. It was revealed that one sex chromosome CNV covering PAR1 (Xp22.33 gain) region was identified in DNA of patient’s testicular tissue (DBN 28) matching with two DECIPHER patient IDs *i.e*., 277458, 290855. This CNV that was clinically reported in patient (DECIPHER patient ID- 290855) as a pathogenic variant may be interpreted as a pathogenic CNV for the SCOS case.

## Conclusion

The present study discussed about genomics of two idiopathic SCOS cases. Both cases were negative for sex chromosome abnormality and Yq microdeletion which directed the study to conduct the SNP microarray for in-depth screening of genomic imbalance. Microarray identified intra-individual genomic variations in different tissues (Blood *vs*. testis) of the recruited SCOS cases. CNVs and LOH related to spermatogenesis identified from two different sample types (Blood *vs*. testicular tissue) were discordant. Hence, the above data suggest that experimental design might be dependent on both sample types to infer viable results. This study should be extended in a larger cohort of patients for validation.
